# Association of the Synapse-Associated Protein 97 (*SAP97*) Gene Polymorphism With Neurocognitive Function in Schizophrenic Patients

**DOI:** 10.3389/fpsyt.2018.00458

**Published:** 2018-09-26

**Authors:** Xusan Xu, Chunmei Liang, Dong Lv, Jingwen Yin, Xudong Luo, Jiawu Fu, Haifeng Yan, Xia Zhou, Zhun Dai, Dongjian Zhu, Susu Xiong, Zhixiong Lin, Juda Lin, Bin Zhao, You Li, Yajun Wang, Guoda Ma, Keshen Li

**Affiliations:** ^1^Department of Neurology, Affiliated Hospital of Guangdong Medical University, Zhanjiang, China; ^2^Guangdong Key Laboratory of Age-Related Cardiac and Cerebral Diseases, Affiliated Hospital of Guangdong Medical University, Zhanjiang, China; ^3^Department of Psychiatry, Affiliated Hospital of Guangdong Medical University, Zhanjiang, China; ^4^Clinical Research Center, Affiliated Hospital of Guangdong Medical University, Zhanjiang, China

**Keywords:** schizophrenia, synapse-associated protein 97 (*SAP97*) gene, L27 domain, rs3915512 polymorphism, neurocognitive function

## Abstract

The *SAP97* gene is located in the schizophrenia susceptibility locus 3q29, and it encodes the synaptic scaffolding protein that interacts with the N-methyl-D-aspartate (NMDA) receptor, which is presumed to be dysregulated in schizophrenia. In this study, we genotyped a single-nucleotide polymorphism (SNP) (rs3915512) in the *SAP97* gene in 1114 patients with schizophrenia and 1036 healthy-matched controls in a Han Chinese population through the improved multiplex ligation detection reaction (imLDR) technique. Then, we analyzed the association between this SNP and the patients' clinical symptoms and neurocognitive function. Our results showed that there were no significant differences in the genotype and allele frequencies between the patients and the controls for the rs3915512 polymorphism. However, patients with the rs3915512 polymorphism TT genotype had higher neurocognitive function scores (list learning scores, symbol coding scores, category instances scores and controlled oral word association test scores) than the subjects with the A allele (*P* = 4.72 × 10^−5^, 0.027, 0.027, 0.013, respectively). Our data are the first to suggest that the *SAP97* rs3915512 polymorphism may affect neurocognitive function in patients with schizophrenia.

## Introduction

Neurocognitive impairment has been described as a core manifestation of schizophrenia, with the impairments occurring mainly in memory, attention, and abstract thinking, etc., and at least two of these cognitive deficits occur in up to 70% of patients (1). The *SAP97* gene encodes the multidomain scaffolding proteins, which are abundantly expressed in neuronal synapses (2) and interact with a variety of neurotransmitter receptors, including NMDAR (3), α-amino-3-hydroxy-5-methyl-4-isoxazolepropionic acid receptor (AMPAR) (4) and serotonin receptor (5-HTR) (5). Because disturbed neurotransmission has been involved in the pathophysiology of impaired cognitive function in schizophrenia (6), the SAP97 proteins that interact with these receptors may also be associated with schizophrenia.

The *SAP97* gene is located in locus 3q29, where there are a significant excess of deletions in schizophrenia patients, and its variants conferred a 17-fold increase in risk for schizophrenia (7). MacKenzie et al. found in rat hippocampal slice cultures that *SAP97* overexpression can drive NMDARs to synapses and enhance NMDA receptor excitatory postsynaptic currents (EPSCs) (2), and knockdown of *SAP97* in another study was found to reduce the expression of AMPAR at the synaptic surface and inhibited NMDA and AMPA EPSCs (8). Moreover, significant reduction of the expression level of the SAP97 proteins have been shown in postmortem brain tissues from schizophrenic patients (9). Therefore, we speculated that the *SAP97* gene is a reasonable candidate gene for schizophrenia.

**Figure 1:**
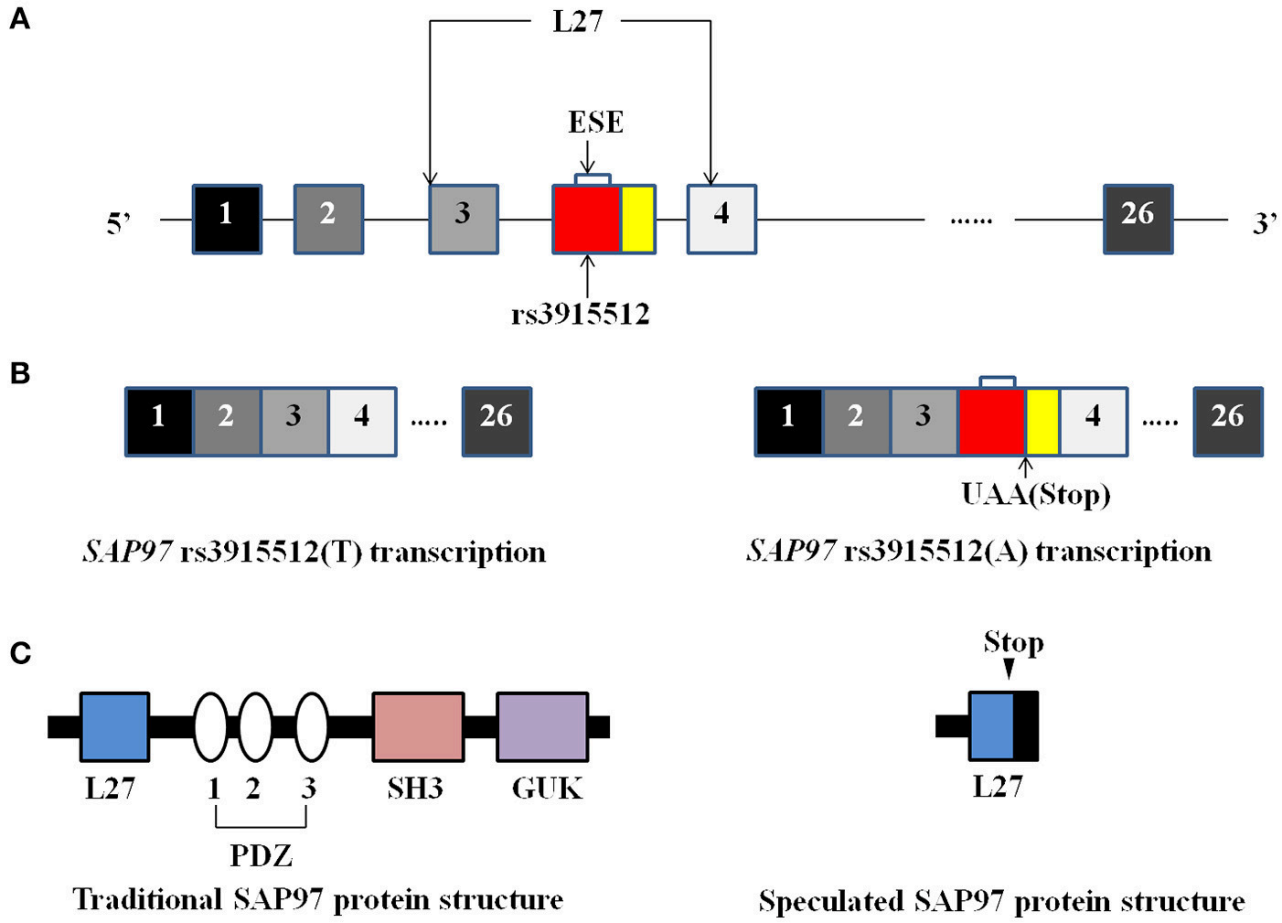
The structure of the human SAP97 gene, transcripts and proteins. **(A–C)** Schematic representation of the structure of the human SAP97 gene **(A)**, transcripts **(B)**, and protein **(C)**. When the SNP rs3915512 consists of the T allele, the SAP97 gene sequence does not meet the exonic splicing enhancer (ESE) consensus and the SAP97 transcript sequence and protein structure was shown on the lower left side of the figure. When the SNP consists of the A allele, an extra exon might insert the transcript because of the ESE consensus. Due to the stop codon in the extra exon, the splicing variant will truncate the SAP97 protein which was shown on the lower right side of the figure. [This figure was improved on the basis of the study by Uezato et al. (10)].

The single nucleotide polymorphism (SNP) rs3915512 has been identified from the region of the *SAP97* gene that encodes the L27 domain at the N-terminal region of the SAP97 protein (10). Uezato et al. found that the T>A variation of the rs3915512 polymorphism would meet the exonic splicing enhancer (ESE) consensus, including the stop codon, which would lead to a premature termination of translation (10) and might have significant effects on the structure of *SAP97* (Figure 1). In addition, studies have shown that changing the structure of SAP97 would alter the binding affinity with its ligands and affect the transport of glutamate (11), which has been identified to play a key role in schizophrenia (6). A recent Japanese case-control study provided evidence of the relationship between the rs3915512 polymorphism of the *SAP97* gene and schizophrenia (12). However, *SAP97* gene polymorphisms have not been studied in schizophrenia patients of the Han Chinese population. Based on the potential role of *SAP97* in the pathogenesis of schizophrenia, the aim of this study was to investigate the association between the rs3915512 polymorphism in the *SAP97* gene and schizophrenia in the Han Chinese population.

## Materials and methods

### Subjects

In this study, 1,138 unrelated patients with schizophrenia (701 males and 413 females, mean age = 34.33 ± 13.56 years) and 1,036 healthy controls (618 males and 418 females, mean age = 34.31 ± 9.43 years) were recruited from the Department of Psychiatry and the Health Examination Center of the Affiliated Hospital of Guangdong Medical University, respectively. All involved subjects were Han Chinese. All patients were diagnosed by at least two well-trained senior psychiatrists according to the Diagnostic and Statistical Manual of Mental Disorders V (DSM-V) criteria and were assessed clinical psychiatric symptoms by using Positive and Negative Symptom Scale (PANSS) (13). Meanwhile, all patients lacked histories of neurological diseases, organic brain injury, mental retardation, alcohol or drug abuse, metabolic disorders and autoimmune illnesses, and none of the healthy controls had substance abuse (excluding nicotine dependence), family histories (first-degree relatives) of psychiatric diseases or severe somatic diseases. Additionally, the present study was approved by the Ethics Committee of the Affiliated Hospital of Guangdong Medical University. All participants gave informed and written consent to take part in the study.

### Genotyping

Genomic DNAs were extracted from EDTA-treated peripheral blood by using a TIANamp Blood DNA Kit (Tiangen Biotech, Beijing, China). As described previously (14), a total of 2,150 individuals were genotyped for the rs3915512 polymorphism of the *SAP97* gene through the imLDR technique (Genesky Biotech, Shanghai, China). The polymerase chain reaction (PCR) primers used for the rs3915512 polymorphism were 5′-TGTTCAGGT GCATCAAGTGGTCTTTACA-3′ (forward primer) and 5′-CTT CAGTAACTTCCAGTCAGATATGGCCT-3′ (reverse primer). The allele-specific probes were as follows: rs3915512RA: 5′-TAC GGTTATTCGGGCTCCTGTCAGTCAGATATGGCCTTACAT CTATCTGTTCAT-3′, rs3915512RP: 5′-AGAATAATTGTTGGT GTGATTTGAAGACTACTTTTTTTTTTTTTTTTTTT-3′, rs3 915512RT: 5′-TTCCGCGTTCGGACTGATATCAGTCAGATAT GGCCTTACATCTATCTGTTCAA-3′.

### Neurocognitive function assessment

The neurocognitive function of the patients was evaluated via the Brief Assessment of Cognition in Schizophrenia (BACS) because of the credible relationship between cognition and the performance in several BACS subtests: working memory (digit sequencing task), verbal memory (list learning), motor speed (token motor task), reasoning and problem solving (Tower of London), attention and processing speed (Symbol coding), semantic and letter fluency (category instances and controlled oral word association test) (15). More details and the specific procedures of the BACS assessment are available in the report by Keefe et al. (15) and Keefe et al. (16).

### Statistical analyses

Quantitative data with normal distributions were expressed as the means ± standard deviations (SD) and the two different groups were compared by using Student's *t*-tests. The Hardy-Weinberg Equilibrium (HWE) and the genotype and allele distributions were assessed with Pearson's Chi-square test. All of the statistical analyses were performed by using SPSS 21.0 software, and statistical significance was defined as *P* < 0.05. Power calculations were performed by using PS -Power and Sample Size Calculation 3.1.2 software (Designed by William D. Dupont, USA).

## Results

### Association study of SNP (rs3915512) and schizophrenia

The genotype distribution of the SNP (rs3915512) reached HWE in both patients and controls (*P* = 0.459 and 0.998, respectively). Age and gender of the healthy controls matched well with the patients with schizophrenia (*P* = 0.964 and 0.119, respectively). In the current study, the frequency of the TT, TA, and AA genotypes was 50.1% (*n* = 558), 42.0% (*n* = 468), and 7.9% (*n* = 88), respectively, in the schizophrenic patients. Additionally, the frequency of these genotypes in the controls was 51.6% (*n* = 535), 40.4% (*n* = 419), and 7.9% (*n* = 82), respectively. Our data revealed that, for the rs3915512 polymorphism, there was no significant difference in the genotype and allele frequency between the patients and the controls (*P* > 0.05). Moreover, no significant differences were detected between the patients and the controls in the gender-stratified analysis (*P* > 0.05) (Table 1).

**Table 1:** Genotyping and allele distribution of rs3915512 on SAP97gene in Chinese controls and patients with schizophrenia.

	**Totel**			**Male**			**Female**		
**dbSNPID**	**Patient**	**Control**	***p*-value**	**Patient**	**Control**	***p*-value**	**Patient**	**Control**	***p*-value**
	***n* = 1114(%)**	***n* = 1036(%)**	**OR(95%CI)**	***n* = 701(%)**	***n* = 618(%)**	**OR(95%CI)**	***n* = 413(%)**	***n* = 418(%)**	**OR(95%CI)**
**rs3915512**
**GENOTYPE**									
TT	558(50.1)	535(51.6)	0.751^a^	345(49.2)	322(52.1)	0.325^a^	213(51.6)	213(51.0)	0.114^a^
TA	468(42.0)	419(40.4)		288(41.1)	249(40.3)		180(43.6)	170(40.7)	
AA	88(7.9)	82(7.9)		68(9.7)	47(7.6)		20(4.8)	35(8.4)	
TA+AA	556(49.9)	501(48.4)	0.472^b^	356(50.8)	296(47.9)	0.295^b^	200(48.4)	205(49.0)	0.859^b^
**ALLELE**									
T	1584(71.1)	1489(71.9)	1.000(reference)	978(70.0)	893(72.2)	1.000(reference)	606(73.4)	596(71.3)	1.000(reference)
			0.577			0.160			0.345
A	644(28.9)	583(28.1)	0.963(0.844–1.099)	424(30.0)	343(27.8)	0.886(0.748–1.049)	220(26.6)	240(28.7)	1.109(0.895–1.375)

*OR, odds ratio; 95%CI, 95% confidence interval*.

a *Global test for the three different genotypes*.

b *Calculations were performed, TA+AA vs. TT*.

Power analysis revealed that, with our study sample and assuming a risk allele frequency of 28.1%, we would have 100.0% power to detect a genotype-relative risk with an odds ratio of 1.5 at the 0.05 level. However, the power was 29.4% for an odds ratio of 1.1 at a significance level of 0.05.

Our study further analyzed the distribution of clinical characteristics in 977 patients among different genotypes, but there were no significant differences in the age of onset, duration of illness, PANSS score or family psychotic history (Table 2).

**Table 2:** Clinical characteristics of the patients with schizophrenia and distribution by genotypes of the SNP.

**Parameters**	**rs3915512**		
	**TT *n* = 493**	**TA+AA *n* = 484**	***p*-value**
Age at onset(years)	24.71 ± 9.45	25.27 ± 10.18	0.371
Duration of illness(years)	10.19 ± 10.08	9.28 ± 9.38	0.144
Years of education(years)	9.24 ± 3.22	9.49 ± 3.20	0.215
PANSS total score	77.77 ± 19.34	77.07 ± 19.48	0.572
P subscore	21.40 ± 7.63	21.63 ± 7.25	0.617
N subscore	18.35 ± 8.95	17.80 ± 8.49	0.319
G subscore	36.51 ± 9.44	36.09 ± 10.10	0.505
Family psychotic history	66(13.4%)	64(13.2%)	0.940
Age at onset(years)			
<18	89(18.1%)	98(20.2%)	0.383
≥18	404(81.9%)	386(79.8%)	

*Values are the mean ± SD; PANSS, Positive and Negative Syndrome Scale*.

### Neurocognitive function analysis

By using BACS, we analyzed neurocognitive function in 359 clinically stable patients and found that the 3915512 polymorphism TT genotypes had higher list learning scores, symbol coding scores, category instances scores and controlled oral word association test scores than the A allele carrier subjects (*P* = 4.72 × 10^−5^, 0.027, 0.027, and 0.013, respectively) (Table 3).

**Table 3:** Neurocognitive functions of the patients with schizophrenia and distribution by genotypes of the SNP.

**Parameters**	**rs3915512**		
	**TT *n* = 177**	**TA+AA *n* = 182**	***p*-value**
**BACS**			
Working memory	17.04 ± 8.97	15.38 ± 8.87	0.079
Semantic fluency	31.16 ± 12.14	28.27 ± 12.39	**0.027**
Letter fluency	10.69 ± 5.97	9.20 ± 5.29	**0.013**
Verbal memory	26.46 ± 15.27	20.38 ± 12.49	**4.72** × **10**^−5^
Motor speed	50.68 ± 17.45	50.01 ± 17.00	0.712
Reasoning and problem solving	8.06 ± 6.10	7.44 ± 6.71	0.359
Attention and processing speed	23.25 ± 13.20	20.13 ± 13.38	**0.027**

*Values are the mean ± SD; BACS, Brief Assessment of Cognition in Schizophrenia. The bold values mean statistically significant*.

## Discussion

The crucial role of neurotransmitters receptor dysfunction, including NMDAR (6, 17), AMPAR (18), and 5-HTR (19), in the development of schizophrenia has been widely accepted. Therefore, the hypothesis that the SAP97 protein, which can interact with these neurotransmitter receptors (3–5), may also play a role in schizophrenia is credible. The signal pathways that include SAP97 are related to long-term potentiation (LTP), potassium channels, and glutamate transport, all of which are associated with learning, cognition and synaptic formation and are impaired in schizophrenia (2, 20). In addition, genome-wide analyses of the copy number variations revealed a microdeletion of the *SAP97* gene in schizophrenia (7, 21). Moreover, Toyooka et al. have found a significant reduction of the expression level of SAP97 protein in postmortem brain tissues from schizophrenic patients (9). Therefore, we speculated that *SAP97* is a reasonable candidate gene for schizophrenia.

**Figure 2:**
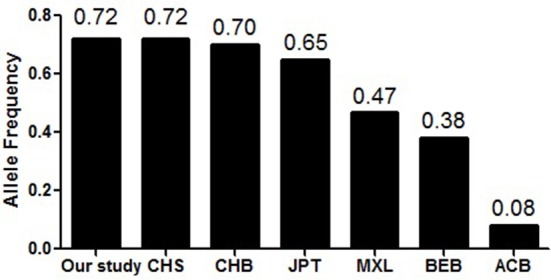
Frequency distribution of the “T” allele of the rs3915512 in different ethnic groups. China. JPT, Japanese in Tokyo, Japan; MXL, Mexican ancestry from Los Angeles USA; BEB, Bengali from Bangladesh;ACB, African Carribbeans in Barbados. (This figure was based on the 1000 Genomes Project).

In this study, we have assessed the potential association of the rs3915512 polymorphism of the *SAP97* gene with schizophrenia. However, the results revealed that there was no significant difference in genotype and allele distributions between the patients with schizophrenia and the healthy controls (*P* > 0.05), which was inconsistent with the results of a previous Japanese cohort study from the Hondo area by Uezato et al. (12). Moreover, the allelic frequencies of rs3915512 in the present study (A:T = 0.29: 0.71 in the cases and A:T = 0.28:0.72 in the controls), were markedly different from those in the Japanese Hondo cohort from a previous study (A:T = 0.63:0.37 in the cases and A:T = 0.66: 0.34 in the controls) (12). This indicates that racial heterogeneity may exist for the distribution of the *SAP97* polymorphism (Figure 2). In the same ethnicity, the allele frequencies of rs3915512 in our cohort from Zhanjiang were similar those of the Chinese population in Beijing (A:T = 0.30:0.70) and were exactly the same as those of the population in southern China (A:T = 0.28:0.72), based on the 1000 Genomes Project (https://www.ncbi.nlm.nih.gov/variation/tools/1000genomes/). Therefore, our data may partly represent the Chinese Han population, and in order to increase the representation of our data, further information extracted from the Han population in other regions of China is necessary.

Studies have shown that schizophrenia is a polygenic inherited disease with a variety of phenotypes (22), and *SAP97* may only affect some of the features of schizophrenia. Therefore, we further analyzed the distribution of clinical manifestations and neurocognitive functions in patients with different genotypes. To the best of our knowledge, this is the first study to investigate the association of the *SAP97* genetic polymorphism with the clinical manifestations and neurocognitive functions of schizophrenia. Although no significant differences were found in the age of onset, duration of illness, PANSS score or family psychotic history, the neurocognitive function scores in patients with the TT genotype were all higher than those of the A allele carriers, and our data demonstrated a significant association between the A allele carriers and lower scores in list learning (*P* = 4.72 × 10^−5^) for verbal memory, in symbol coding (*P* = 0.027) for processing speed, in category instances (*P* = 0.027) and controlled oral word association test (*P* = 0.013) for verbal fluency in our study cohort. Therefore, we speculated that the A allele of the rs3915512 polymorphism may be a detrimental factor for schizophrenia that affects the neurocognitive function of patients with schizophrenia.

The SNP rs3915512 is located in an important region of the *SAP97* gene where it is transcribed into a portion of the *SAP97* mRNA that is further translated into a part of the L27 domain (10). As an assembly center for large proteins (23), the L27 domain can mediate the multimerization of scaffold proteins that are encoded by the *SAP97* gene (24). Although it only interacts with multimeric scaffold proteins, NMDAR can trigger the recruitment of AMPARs into the synaptic membrane and thus plays a key role in synaptic plasticity (25), which is directly related to cognitive function (26). Therefore, we speculated that SAP97 acts as a bridge and plays an important role in neurocognitive function. We hypothesized that in addition to changing the nucleotide sequence of the *SAP97* gene, differences in the SNP distribution pattern might also result in changes to the mRNA sequence and/or the protein structure of SAP97 (3). A study of the *SAP97* gene found that the variation of the rs3915512 polymorphism might truncate the part of the SAP97 protein (10). And Changes of SAP97 protein structure would inevitably affect the interactions of SAP97 protein with various neurotransmitter receptors, which may lead to disorders in neurotransmitter delivery. Based on our results, this seems to be a reasonable argument, but it still needs further research and verification.

There are several limitations in our current study. First, power analyses revealed that, with our study sample and assuming a risk allele frequency of 28.1%, we would have 100.0% power to detect a genotype relative risk with an odds ratio of 1.5 at the 0.05 level. However, the power was 29.4% for an odds ratio of 1.1 at a significance level of 0.05. Therefore, false negative results cannot be ruled out. The current study is limited in that, despite the influence of the genotype of SNP rs3915512 on neurocognitive function in schizophrenia, the specific mechanism is unclear. Moreover, our current results were limited to the Han Chinese population and we also did not further clarify the relationship between other SNPs in the *SAP97* gene and schizophrenia.

In summary, our study first revealed that the *SAP97* gene rs3915512 polymorphism may be associated with the neurocognitive impairment that results from schizophrenia in a Chinese Han population, and the A allele of the rs3915512 polymorphism may be a detrimental factor for schizophrenia patients.

## Ethics statement

This study was carried out in accordance with the recommendations of the Ethics Committee of the Affiliated Hospital of Guangdong Medical University with written informed consent from all subjects. All subjects gave written informed consent in accordance with the Declaration of Helsinki. The protocol was approved by the Ethics Committee of the Affiliated Hospital of Guangdong Medical University.

## Author contributions

KL, GM, YW, JL, BZ, YL, and ZL conceived and designed the experiments and revised the manuscript. JY, XL, JF, and HY did genetic analyzes. XZ, ZD, DZ, and SX collected the cognitive and clinical data. XX, CL, and DL analyzed and interpreted the data and drafted the manuscript. All authors were involved in the revision of the manuscript.

### Conflict of interest statement

The authors declare that the research was conducted in the absence of any commercial or financial relationships that could be construed as a potential conflict of interest.
